# Kidney Transplantation and Cellular Immunity Dynamics: Immune Cell Alterations and Association with Clinical and Laboratory Parameters

**DOI:** 10.3390/jcm13175093

**Published:** 2024-08-27

**Authors:** Lampros Vagiotas, Georgios Lioulios, Manolis Panteli, Konstantinos Ouranos, Aliki Xochelli, Efstratios Kasimatis, Vasiliki Nikolaidou, Margarita Samali, Maria Daoudaki, Georgios Katsanos, Nikolaos Antoniadis, Georgios Tsoulfas, Maria Stangou, Asimina Fylaktou

**Affiliations:** 1 Department of Transplant Surgery, General Hospital Hippokratio, 54642 Thessaloniki, Greece; lampisv@yahoo.gr (L.V.); katsanosg@auth.gr (G.K.); nikanton@auth.gr (N.A.); tsoulfas@auth.gr (G.T.); 2 Department of Nephrology, 424 General Military Hospital of Thessaloniki, 56429 Thessaloníki, Greece; 3 1st Department of Nephrology, General Hospital Hippokratio, 54642 Thessaloniki, Greece; pantelimanolis@gmail.com (M.P.); frasci@outlook.com.gr (E.K.); mstangou@auth.gr (M.S.); 4 Department of Medicine, Houston Methodist Research Institute, Houston, TX 77030, USA; kostassky@hotmail.com; 5 National Peripheral Histocompatibility Center, Department of Immunology, General Hospital Hippokratio, 54642 Thessaloniki, Greece; aliki.xochelli@gmail.com (A.X.); basoniko@hotmail.com (V.N.); margaritasamali@hotmail.com (M.S.); fylaktoumina@gmail.com (A.F.); 6 School of Medicine, Aristotle University of Thessaloniki, 45636 Thessaloniki, Greece; daoudaki@auth.gr

**Keywords:** cold ischemia time, kidney transplantation, natural killers, regulatory T cells, T cells

## Abstract

**Background/Objectives**: The purpose of this study was to evaluate numerical changes in immune cells after successful kidney transplantation and associate their recovery with clinical and laboratory factors. **Methods**: In 112 kidney transplant recipients, we performed flow cytometry to evaluate counts of CD4+, CD8+, and regulatory T cells (Tregs), as well as natural killer (NK) cells, before kidney transplantation (T0) and three (T3), six (T6), and twelve (T12) months later. The results were associated with the recipient’s age, cold ischemia time (CIT), the type of donor, dialysis method and vintage, and graft function in one year. **Results**: Total and CD8+ T cell counts increased gradually one year post transplantation in comparison with pre-transplantation levels, whereas the number of CD4+ T cells and Tregs increased, and the number of NK cells decreased in the first three months and remained stable thereafter. The recipient’s age was negatively correlated with total, CD4+, and Treg counts at T12, whereas CIT affected only total and CD4+ T cell count. Moreover, recipients receiving kidneys from living donors presented better recovery of all T cell subsets at T12 in comparison with recipients receiving kidneys from cadaveric donors. Patients on peritoneal dialysis had increased numbers of total and CD8+ T cells, as well as NK cells. Finally, estimated glomerular filtration rate was positively correlated with Treg level and potentially CD4+ T cells one-year post transplantation. **Conclusions**: Successful kidney transplantation results in the recovery of most T cell subsets. Lower recipient age and better graft function contribute to increased T cell counts, whereas donor type and dialysis modality are the most important modifiable factors for optimal immune recovery.

## 1. Introduction

Phenotypic and functional changes of the immune system, such as lymphopenia and T lymphocyte subset alterations, including reduced levels of CD4+ and CD8+ T cells, as well as a reduced CD4+/CD8+ ratio, have been described in patients with chronic kidney disease [1,2]. Kidney transplantation is the treatment of choice for patients with end-stage kidney disease (ESKD) as it improves the recipient’s survival and quality of life, absolves patients from dialysis, optimally substitutes kidney function, and promotes the reinstatement of the patient’s immune profile [3,4]. In patients undergoing kidney transplantation, estimated glomerular filtration rate (eGFR) is a primary marker for the clinical monitoring of allograft function and long-term transplant survival [5,6].

Transplant rejection is caused by the activation of T lymphocytes that mount a coordinated immune response against the graft, which involves both the adaptive and the innate arms of the immune system [7,8]. CD4+ T cells play a central role in the induction of different effector mechanisms of allograft rejection, while CD8+ T cells define a population of T cells exhibiting direct cytotoxic action [7]. Regulatory T cells (Tregs) derived from activated T cells are the most potent and well-defined regulatory T cell subpopulation, capable of exerting a variety of actions on effector T cells, especially inhibition of cell proliferation and cytokine and antibody production [9]. Another cell type, CD56dim/CD16+ natural killer (NK) cells, has been described as a major NK cell subset that could be involved in the pathogenesis of renal rejection, either antibody- or T cell-mediated [10].

Although several studies have shown that renal transplantation affects certain T cell populations, both in the short and in the long term, it is not clear yet whether and under which circumstances it can fully restore the main lymphocyte subsets. In the present study, we aimed to evaluate the amelioration of the immune cell phenotypic profile over the course of time, following successful renal transplantation. To this end, we investigated changes in T cell phenotype three, six, and twelve months after transplantation in comparison with the pre-transplant period and associated our findings with potential contributing factors, such as eGFR and peri-transplantation circumstances.

## 2. Materials and Methods

### 2.1. Study Design

This is a prospective, observational study conducted in the Department of Renal Transplantation, Hippokratio General Hospital, Thessaloniki, Greece, between January 2020 and January 2023. All adult patients receiving ABO-compatible kidney transplantation with negative complement-dependent crossmatch were eligible for the study, including transplants from either cadaveric or living donors. The day of transplantation was considered the day of enrolment in the study. Demographic and clinical data from recipients and donors were recorded on the day of admission. For ESKD patients on dialysis, a dialysis modality was also recorded. Patients with a history of recent (less than 3 months) serious infection, recent malignancy (less than 5 years), active autoimmune or inflammatory disease, hematological disorders, or immunosuppressive treatment during the last 12 months prior to kidney transplantation were excluded from the study.

For the analysis of pre-transplantation immune phenotype (T0), blood samples were obtained as described in the relevant section before the administration of any immunosuppressive treatment. During the immediate post-transplantation period, renal function, medication and potential side effects, cold ischemia time (CIT), delayed graft function (DGF), acute rejection episodes, infections, and deaths were recorded. Immunosuppressive treatment included induction with basiliximab according to the local protocol (20 mg IV one hour before transplantation and 20 mg on day four, accompanied by two doses of 500 mg methylprednisolone IV the night before and one hour before transplantation) and maintenance treatment with methylprednisolone (initiation with 125 mg IV the first day after transplantation and then 16 mg pos until the 14th day and then tapering by 4 mg after on days 14, 28, and 42), tacrolimus (plasma levels 6–8 ng/mL for the first year), and mycophenolate mofetil (2 g per day until day 14 and then 1 g per day). Patients with a rejection episode received anti-thymocyte globulin, as per protocol. Following discharge from the hospital, all patients were regularly followed up at the outpatient clinic of our center. The immune profile of the patients was further analyzed three (T3), six (T6), and twelve (T12) months following transplantation, as described below. At the same time points, renal graft function was evaluated via eGFR, with the CKD-EPI 2009 equation for creatinine. Moreover, infections and deaths and de novo donor-specific antibodies were recorded during the follow-up period. Deceased patients, patients with graft failure, and patients not compliant with treatment instructions were eventually excluded from the final analysis.

All participants provided written informed consent before their enrollment. The study was approved by the Ethics Committee of Aristotle Hippokration of Thessaloniki (No 2348/24-11-2019) and conducted according to the principles of the Declaration of Helsinki (2008 Amendment).

### 2.2. Flow Cytometry

**Figure 1:**
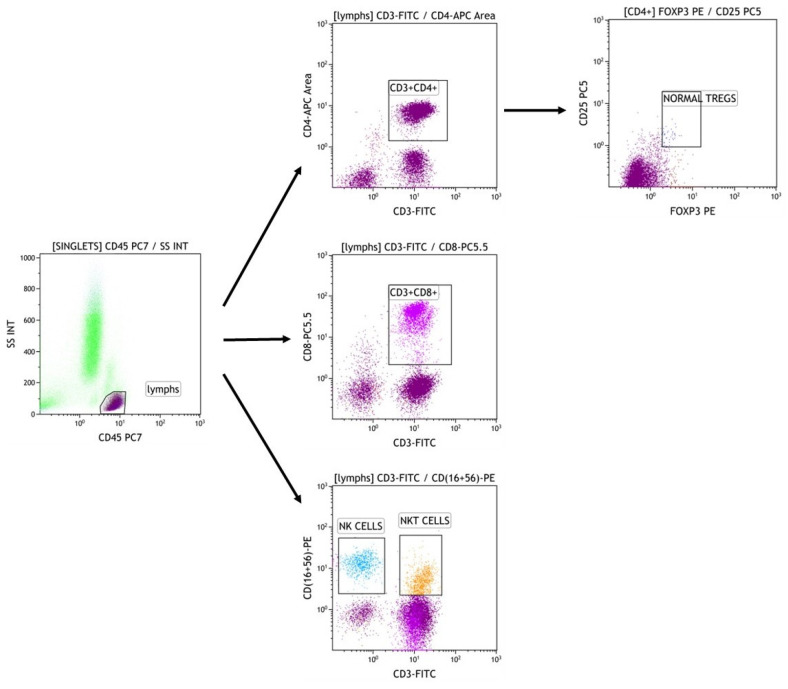
Gating strategy of T cell subsets.

Whole blood samples were obtained from participants at the aforementioned time points (T0, T3, T6, and T12) in K2EDTA tubes and were immediately transferred to the laboratory, where they were kept at room temperature until processing, for no longer than 12 h. The blood samples were stained with the following conjugated antibodies (manufactured by Beckman Coulter, Brea, CA, USA): CD45 PC7 (Clone J33), CD3 FITC (Clone UCHT1), CD16-CD56 PE [CD16: Clone 3G8, CD56: Clone N901(NKH-1)], CD4 APC (Clone 13B8.2), CD8 PC5.5 (Clone B9.11), CD4 FITC (Clone SFCI12T4D11), CD25 PC5 (Clone B1.49.9), and FOXP3 PE (Clone 259D, intracellular staining). Immune phenotype was assessed with a cell counter (Navios EX Flow Cytometer, Beckman Coulter) according to the manufacturer’s recommendations, and cell subsets were determined by the expression of surface markers as follows: CD4+ T cells (CD3+CD4+), CD8+ T cells (CD3+CD8+), NK cells (CD3-CD16+CD56+). Tregs were defined as (CD4+CD25+FOXP3+). The gating strategy is shown in Figure 1.

### 2.3. Statistics

Given the fact that our center performed an average of 55 kidney transplantations per year in the 3 years before study initiation, our sample size was calculated to be 116 recipients for a 5% margin of error and 95% confidence interval. The significance level was set at 5%. Continuous variables were tested for normality with the Shapiro–Wilk test and were reported as the median (25th–75th percentile). Non-continuous variables were reported as absolute value and relative frequency (N, %). A comparison of continuous variables was performed with non-parametric tests (Mann–Whitney U-test for two non-related populations and the Friedman test for multiple related variables). After logarithmic transformation, two-way repeated measures ANOVA was used to investigate the impact of potential contributing factors to changes in lymphocytic populations with time. Spearman’s rank correlation coefficient was used to estimate the correlation between age and antibody titers. For the correlation to be considered significant, the R-value should be greater than the critical value for the corresponding degrees of freedom. Finally, multivariate linear regression was used to assess the effects of multiple factors on immune cell variations. Statistical analysis was performed with the Statistical Package for the Social Sciences (SPSS), version 28 for Windows, IBM, Armonk, New York, NY, USA (SPSS 28.0).

## 3. Results

### 3.1. Patient Characteristics

One hundred and nineteen patients were originally eligible for the study, of whom four deceased patients, one patient with graft failure, and two non-compliant patients were excluded from the final analysis. One hundred and twelve 18–72-year-old recipients were eventually included in the study, with a median age of 48.5 (39–57) years. Of them, N = 83 (74.1%) received a kidney from a cadaveric donor, whereas N = 29 (25.9%) received a graft from a living donor. The characteristics of the recipients and donors are depicted in Table 1. The most common cause of ESKD was primary glomerulopathies, followed by polycystic kidney disease. Diabetic kidney disease and hypertensive nephrosclerosis were not as common among the patients included, potentially due to comorbidities constituting contraindications to kidney transplantation. However, diabetes and hypertension were underrepresented as causes of ESKD in our study population.

### 3.2. Changes in Immune Cell Phenotype during Follow-Up

**Figure 2:**
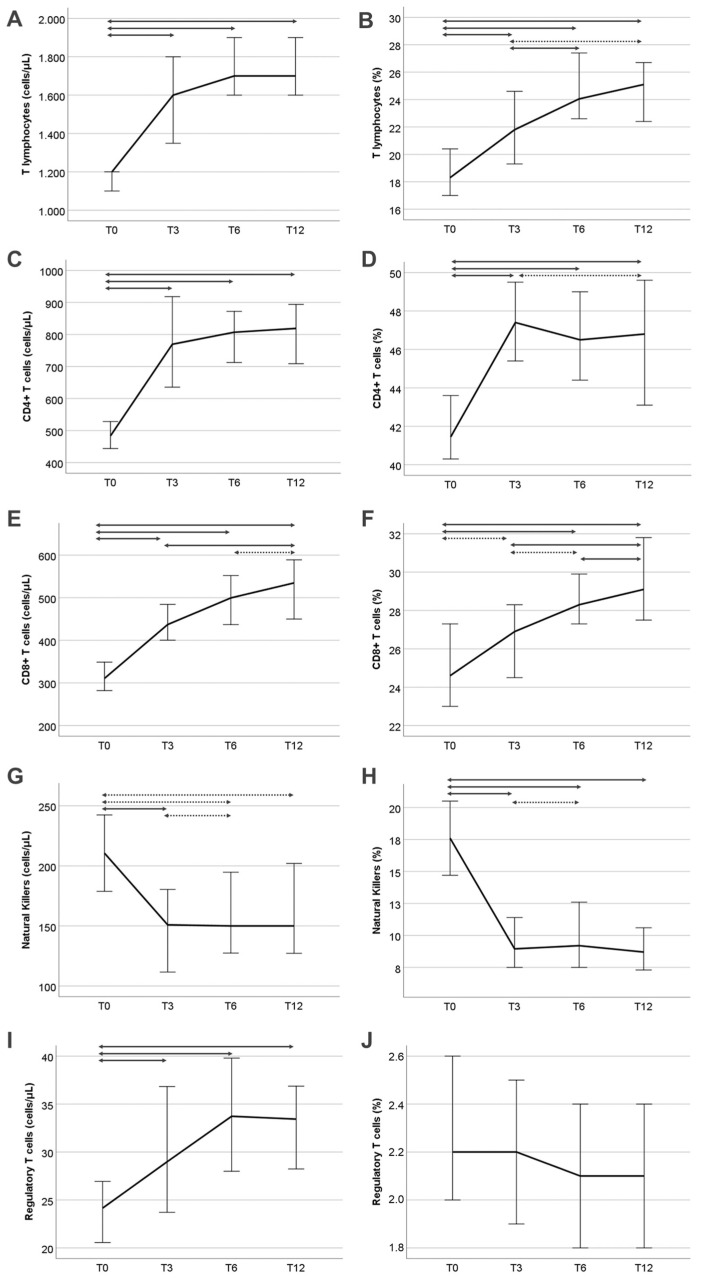
Changes in absolute count (**A**,**C**,**E**,**G**,**I**) and percentage (**B**,**D**,**F**,**H**,**J**) of cell subsets during the 12-month follow-up of total and percentage T cells, CD4+ cells, CD8+ cells, natural killer cells, and regulatory T cells, respectively. Double arrows denote statistical significance; straight lines: *p* < 0.008; dotted lines 0.008 < *p* < 0.05 (not significant after Bonferroni correction).

T lymphocytes and their subpopulations showed significant changes during the 12-month follow-up (Table 2). The most prominent changes were observed during the first three months (from T0 to T3) during which all of the studied subsets showed a significant increase in their total count, except for NK cell count which decreased. The decrease in NK cells was more prominent in their proportion, which decreased significantly to more than half relative to the baseline level. After T3, total T lymphocytes and CD8+ T cell count followed a less accelerated but constant increase up to T6 and T12. However, CD4+ T cell, NK cell, and Treg count did not change significantly from T3 to T6 and T12 (Table 2). An increase in CD4+ T cell and Treg count was also observed from T3 to T6, but it was not statistically significant. Of note, the proportion of Tregs did not change at all during the follow-up. Post hoc analysis of lymphocyte subset changes during the first 12 months post-transplantation is provided in Supplementary Tables S1 and S2 and Figure 2.

### 3.3. Effect of Clinical and Laboratory Parameters in Adaptive Immunity during Follow-Up

#### 3.3.1. Role of Peri-Transplantation Circumstances

Several perioperative factors had a significant impact on immune cell subpopulations one year after successful renal transplantation. The age of the recipient at transplantation negatively affected T cell recovery one year after transplantation (r = −0.3, *p* = 0.002). However, not all T cell subsets were affected equally, as significance remained for CD4+ T cells (r = −0.34, *p* < 0.001) and Tregs (r = −0.31, *p* = 0.001), but not for CD8+ T cells or NKs. Of note, these associations were observed as early as three months after transplantation and were preserved at the twelve-month time point (Supplementary Table S3).

The effect of CIT was also negative for total T cell count at T12 (r = −0.22, *p* = 0.02) and CD4 T cells (r = −0.26, *p* = 0.009), whereas CD8+ T cells were barely affected by CIT (r = −0.19, *p* = 0.04, marginally significant for degrees of freedom). Tregs and NKs were not affected by CIT (Supplementary Table S1). The observation of the significance of CIT in the recovery of T cell subsets is further supported by the increase in T cell count in patients receiving kidneys from live donors in comparison with patients receiving kidneys from deceased donors at T12. [T cells: 2300 (1600–2900) vs. 1644 (1300–2100) cell/μL, *p* = 0.001, CD4+ T cells: 997 (815–1493) vs. 719 (527–961) cells/μL, *p* = 0.001, CD8+ T cells 650 (463–947) vs. 476 (366–659) cells/μL *p* = 0.007, Tregs: 42 (24–58) vs. 31 (21–41) cells/μL, *p* = 0.04, for live and deceased donors, respectively]. However, CIT possibly only partially explained T cell differences between the two groups as the CD8+ T cell count also increased in live donor recipients, whereas they were not affected by CIT. NK cell count did not differ between the two groups of patients. Preemptive transplantation did not have an impact on T cells or NK counts as no difference was observed between live-donor recipients on dialysis or not (N = 17 and N = 12, respectively). Of note, CIT did not affect the rates of DGF in our population as patients with or without DGF had comparable CIT [16 (0–19) vs. 18.25 (13–21.75) hours, *p* = 0.179]. The difference was not significant even when we removed recipients from live donors from the comparison (CIT = 0 h), [14 (16–18) vs. 18.75 (17.25–22.25) hours, respectively, *p* = 0.163].

Dialysis vintage (DV) negatively affected total T cell count at T12 (*p* = −0.29, r = 0.002), but analysis of the subsets showed that only CD4+ T cells were negatively associated with DV at T12 (r = −0.32, *p* = 0.001), whereas the rest of the subsets were not affected (Supplementary Table S1). The dialysis method had a significant impact on T cell recovery at T12 as patients on peritoneal dialysis (PD) had increased counts of T cells [2500 (1798–3100) vs. 1600 (1300–2126) cells/μL, *p* = 0.005], CD8+ T cells [675 (589–987) vs. 471 (375–678) cells/μL, *p* = 0.01] and NKs [219 (198–467) vs. 148 (101–291) cells/μL, *p* = 0.01] in comparison with hemodialysis (HD) patients. CD4+ T cell and Treg count did not differ according to the dialysis method.

Associations between the recipients’ age, DV, and CIT and total and CD4+ T cell counts at T12 were confirmed using linear univariate regression models (Table 3). However, in multivariate analysis, age was the only significant factor that could predict total and CD4+ T cell count and could explain around 20% of the total variance for both populations at T12 [T cells: partial β = −0.32, 95% CI: −26.5, −6.6, CD4+ T cells: partial β = −0.33, 95% CI: −14.8, −3.9]. NK cells and Tregs were not included in the multivariate analysis as they were affected only by one of the studied factors.

#### 3.3.2. The Role of Renal Function Outcomes after Renal Transplantation

To evaluate the effect of eGFR on immune phenotype recovery, we correlated eGFR at T12 with T cell counts. Moreover, we categorized the patients into two groups according to their eGFR at T12: group 1 comprised patients with eGFR > 50 mL/min/1.73 m^2^ and group 2 comprised patients with eGFR ≤ 50 mL/min/1.73 m^2^, and we compared changes in immune cell counts between the two groups. Graft function as assessed by eGFR, [median eGFR at T12: 63 (53–76) mL/min/1.73 m^2^], significantly affected Treg count restoration. At T12, Treg count was positively correlated with eGFR (r = 0.2, *p* = 0.04). The rest of the T cell subset counts were not associated with eGFR at any time point. Moreover, a comparison between the two groups of patients according to eGFR showed that patients with eGFR > 50 had increased CD4+ T cell counts in comparison with patients with eGFR < 50 [857 (616–1122) vs. 628 (458–893) cells/μL, *p* = 0.03].

**Figure 3:**
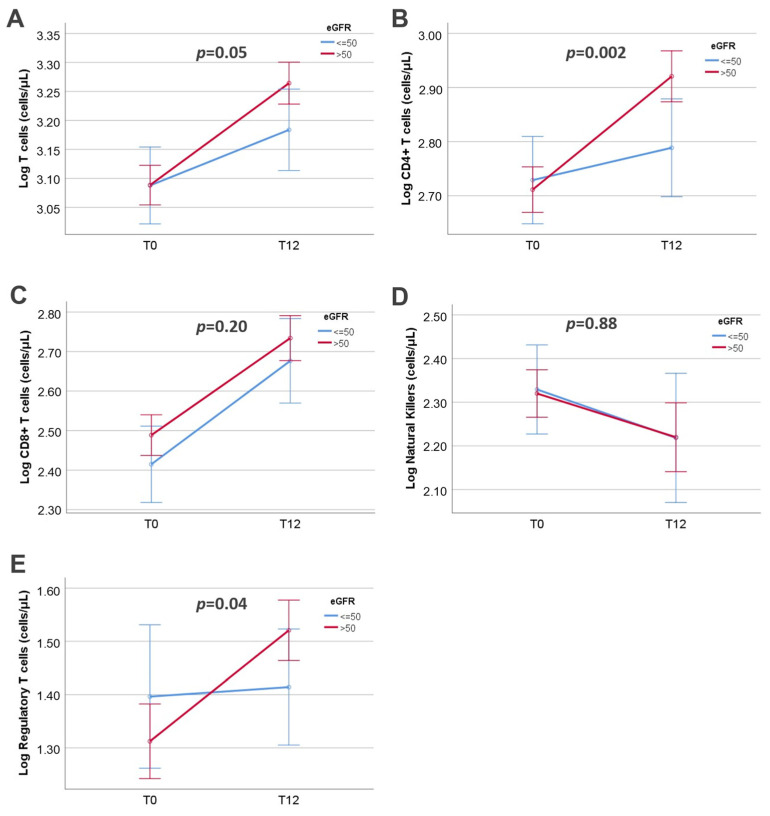
Changes of T cell subset counts from pretransplant levels (T0) to levels 12 months after transplantation (T12), after logarithmic transformation, according to estimated glomerular filtration rate (eGFR) at T12. (**A**). Total T cells, (**B**). CD4+ T cells, (**C**). CD8+ T cells, (**D**). Natural Killer cells, (**E**). Regulatory T cells.

Significant differences were also observed in the magnitude of immune cell recovery during the 12-month period between the two groups of patients, as shown in Table 4. Total T cells and CD8+ T cells increased significantly from T0 to T12 in both patient groups, although to a lesser degree in the low eGFR group. On the other hand, CD4+ T cell and Treg count increased significantly only in the high eGFR patient group, while the increase in these subsets was not significant in the low eGFR group. Finally, the NK count dropped non-significantly in the low eGFR group but increased in the high eGFR group. In two-way repeated measures ANOVA, a significant difference was found in total T cells and CD4+ T cells, including Treg recovery between the two groups of patients (Figure 3).

Finally, six patients had at least one episode of acute rejection during the follow-up period. Although no significant differences were found at T12 in the count of lymphocyte subsets, the sample size was too low to extract safe conclusions.

#### 3.3.3. Role of Lymphocyte Subsets in Outcomes

During the follow-up period, 13 patients were admitted due to infections and were treated with intravenous antibiotics. These patients had significantly lower NK counts at T0 [124 (95–198) vs. 222 (159–326) cells/μL, *p* < 0.001]. This difference was also present at T12 [98 (62–140) vs. 168 (112–305) cells/μL, *p* = 0.04]. Patients with at least one rejection episode did differ in their immune cell counts at T0.

## 4. Discussion

In this prospective, observational study, we evaluated changes in the lymphocytic phenotypic profile of patients after successful kidney transplantation and their association with several peri- and post-transplantation factors. More specifically, we estimated the T cell subset counts, including total, CD4+, CD8+ T cells, Tregs, and NK cells, of the patients at three time points up to 12 months post-transplantation and examined the impact of recipient age, CIT, donor type, dialysis method, and dialysis vintage, as well as parameters during follow up, such as renal graft function.

Lymphocytic phenotype changed radically after transplantation. The absolute count of lymphocytes, CD4+ and CD8+ T cells, and Tregs increased significantly over time after kidney transplantation, while the absolute count of NK cells decreased significantly over the same period. An increase in total and CD4+ cell count four weeks post-transplantation has already been described in a study in 2004 [11]. However, the underlying mechanism is not well understood, as data on the restoration of thymic function are not sufficient [12,13]. Of note, another recent study has shown that 3 years post transplantation, the relative frequencies of CD4+ T cells and Tregs were lower in comparison to ESKD patients or healthy controls, despite the increase in total T cell percentage [14]. The latter study also showed an increase in CD8+ T cell proportion in transplanted patients. Finally, a small study published in 2018 reported no difference in NK cell proportion and count between healthy and transplanted women [15].

Data on the kinetics of Tregs after kidney transplantation remain inconclusive. There might be a preferential expansion of conventional T cells over Tregs, which may have implications for the balance between immune activation and regulation. Low levels of Tregs for six months after kidney transplantation, potentially followed by a gradual recovery to almost basal levels during the first posttransplant year, have been reported, which may be explained by the high burden of immunosuppression in the early post-transplant period [16,17]. Low levels of Tregs might have an important clinical impact, as they have been associated with an increased risk of acute rejection and chronic allograft dysfunction [18,19]. Therefore, strategies to enhance the number or function of Tregs may be beneficial for improving transplant outcomes.

Reinforcing our findings on the impact of recipient age on T cells, a study by Wang et al. reported that in subgroup analysis for age, only patients older than 50 years of age had a significant decrease in T cell proportion, three years after transplantation compared to pre-transplantation levels, but this difference was not significant for CD4+ T cells [14]. However, the same study reported an increase in the proportion of CD8+ T cells in the same patient group. Moreover, in a study by Schaenman et al., younger and older transplanted patients had similar CD4+ and CD8+ T cell percentages three months after transplantation, but only younger patients had significantly increased proportions of naïve CD4+ and CD8+ T cells. Both of the above studies did not examine absolute counts of T cell subsets. These findings underscore the importance of considering the age-related changes in T cell subsets when managing patients undergoing renal transplantation.

CIT is a crucial factor for the outcome of renal transplantation as it affects ischemia–reperfusion injury and inflammatory and immune responses that may impair graft function and survival. T cells are important mediators of the alloimmune response and may also be affected by CIT, as longer CIT has been associated with increased T cell activation, proinflammatory cytokine production, and apoptosis in the early post-transplant period [20]. In our study, CIT was negatively correlated with counts of CD4+ and CD8+ T cells one year after kidney transplantation. CIT can influence the number and function of CD4+ and CD8+ T cells in the graft, as well as their interaction with antigen-presenting cells and other immune cells, and may induce apoptosis, necrosis, or senescence of T cells, reducing their viability and responsiveness [21]. Moreover, CIT can affect the number and function of Tregs depending on the organ type, duration and temperature of ischemia, and preservation solution, reducing the frequency and suppressive capacity of Tregs [21,22]. Increased CIT has been reported to promote NK cell infiltration in the blood vessels and interstitium of kidney tissue, which in turn leads to impaired cytokine production and worse kidney graft outcomes [22,23]. This may increase the long-term risk of infection and malignancy, while, in the short-term, NK cells can also mediate ischemia–reperfusion injury and allograft rejection by producing inflammatory mediators and activating adaptive immune cells [23]. Based on our results, reducing CIT may affect T cell numbers after renal transplantation, which may indirectly affect graft survival. However, we found no association of CIT with Tregs or NK cells.

Donor type significantly influenced the number of CD4+ and CD8+ T cells and Tregs in kidney transplant recipients. Studies have indicated that living donor kidney transplants often result in better immune cell recovery and function, possibly due to the reduced stress on the cells from a shorter preservation time [21,24]. Molecular mechanisms may involve the expression of the Donor Toll-like 4 receptor, which was found to be upregulated in deceased donors as compared to living donor kidneys, and the induction of complement fraction C3 in brain death donors, which was found to be associated with reduced allograft function [25,26].

Dialysis method has been shown to significantly affect the immune system, as patients undergoing PD exhibited an increased number of CD4+ cells and a decreased number of CD8+ T cells compared to HD, before transplantation [11]. However, our results suggest an increase in CD8+ T cell count in PD patients post transplantation in comparison with HD patients. Moreover, Caprara et al. found that Treg count increased in patients on PD, in comparison with patients on HD, one month after transplantation, while the number of CD4+ T cells did not differ between the two patient groups [27]. NK cell count may also be affected by the dialysis method, as, in HD patients, the number and cytotoxicity of NK cells decrease [28]. However, PD patients with prevalent coronary artery disease had elevated NK cell levels [29].

In accordance with our results, Chiu et al. described a reduction in both total T cells and CD4 + cells with longer DV, while also affecting their cellular differentiation, resulting in a reduction in naïve CD4+ and CD8+ T cell numbers but an increase in CD8+ differentiated cells. The authors state that there is a link between the uremic environment that induces chronic inflammation and oxidative stress and the levels of immune cells. Therefore, reducing DV may be beneficial for preserving T cell immunity and improving transplant outcomes.

Estimated GFR appeared to be an important determinant of immune cell recovery one year after transplantation, as it has been positively correlated with the number of NK cells in un-dialyzed non-transplanted CKD patients [30]. NK cells play a pivotal role in immune surveillance in the setting of kidney transplantation. Altered NK function, and potential number, has been associated with infectious complications, but the reduced count is associated with an increased risk of developing non-melanoma skin cancer in these patients [28,31]. In our study, patients with serious infections during the first year after transplantation had significantly reduced NK cell numbers compared to those who did not develop an infection, both before and one year after transplantation. Interestingly, a number of studies have shown that reduced numbers of circulating T cells and impaired proliferative T cell responses persist even after successful kidney transplantation [32,33,34]. Immunosuppressive treatment might play a central role in this. On the other hand, the count of CD4+ T cells may play a role in the long-term survival of the graft [35]. In our study, patients in the low eGFR group had a significantly lower CD4+ T cell count one year after transplantation. However, it cannot be proven whether insufficient graft function did not allow for the full recovery of CD4+ T cell count or vice versa.

We acknowledge some limitations of our study. Even though the examined population was homogeneous, this is a single-center study, and therefore, the generalizability of the results may be limited. Given the complexity of immune interactions between the studied subsets, investigation of the functional characteristics of T cell subsets, such as cytokine production, proliferation, activation markers, and receptor expression, would further enhance the importance of our findings. These processes may vary depending on the type and duration of immunosuppression, antigen exposure, and inflammation. Finally, the study did not take into account the long-term follow-up that is needed to evaluate the clinical implications of the observed changes.

## 5. Conclusions

In conclusion, our study demonstrates that kidney transplantation induces significant changes in T cell phenotypes over time and these changes are influenced by several clinical and laboratory factors. The restoration of T cell phenotypes after kidney transplantation is important for achieving immunological tolerance and preventing chronic allograft dysfunction. However, the dynamics and factors that influence the recovery of different T cell subsets are not fully understood.

## Figures and Tables

**Table 1:** Clinical and demographical characteristics. HD: hemodialysis, PD: peritoneal dialysis, CVA: cardiovascular accident, CD: cadaveric donor, and LD: living donor.

Recipients’ Characteristics	N = 112
Male/Female, N (%)	77/35 (68.8/31.3%)
Age (yrs)	48.5 (39–57)
Primary kidney disease, N (%)	
Nephrosclerosis/hypertension	6 (5.3%)
Primary glomerulopathies	26 (23%)
Diabetes mellitus	6 (5.3%)
Urinary tract infections/stones	6 (5.3%)
Reflux nephropathy	13 (11.6%)
Polycystic kidney disease	24 (21.4%)
Other	16 (14.2%)
Unknown	15 (13.3%)
Dialysis Data, N (%)	
HD/PD	89/11 (79.5/9.8%)
Dialysis vintage (months)	87 (34–127)
Preemptive transplantation	12 (10.7%)
Retransplantation	10 (8.9%)
Transplantation	
Cold ischemia time (hours)	17 (0–19)
Delayed graft function, Ν (%)	20 (17.9%)
Rejection, N (%)	7 (6.3%)
Infections, N (%),	13 (11.6%)
Induction with basiliximab	106 (94.6%)
Treatment with anti-thymocyte globulin, N (%)	17 (15.2%)
Donors’ characteristics	
Male/Female	58/54 (51.8/48.2%)
Age (years)	53 (14–76)
Marginal donor, Ν (%)	6 (5.4%)
Donor cause of death	
Anoxia	4 (3.6%)
CVA	60 (53.6%)
Trauma	19 (17.0%)
Donor relationship, N (%)	
CD/LD	83/29 (74.1/25.9%)

**Table 2:** White cell subset changes at T0, T3, T6, and T12 time points after transplantation.

T Cell Subsets	T0	T3	T6	T12	*p*
T cells (cells/μL)	1200 (1000–1600)	1600 (1200–2300)	1700 (1300–2300)	1700 (1400–2300)	<0.001
T cells (%)	18.3 (14.8–24)	21.8 (16.8–31.5)	24 (20.2–31.6)	25.1 (20.3–31.6)	<0.001
CD4+ (cells/μL)	483 (384–707)	769 (526–1142)	807 (574–1116)	819 (600–1107)	<0.001
CD4+ (%)	41.4 (36.1–48.7)	47.4 (40.5–53.4)	46.5 (40.3–53.4)	46.8 (38.3–53.9)	<0.001
CD8+ (cells/μL)	310 (215–416)	437 (291–633)	499 (341–648)	534 (387–743)	<0.001
CD8+ (%)	24.6 (20.4–30.1)	26.9 (21.7–32)	28.3 (24.1–34)	29.1 (24–38)	<0.001
NK cells (cells/μL)	210 (152–307)	151 (86–245)	150 (105–270)	150 (106–299)	<0.001
NK cells (%)	17.6 (11.8–25.2)	8.9 (5.4–14.9)	9.2 (6.1–16.5)	8.7 (6–15.2)	<0.001
Tregs (cells/μL)	24 (15–33)	29 (18–51)	33 (24–48)	33 (21–48)	<0.001
Tregs (%)	2.2 (1.4–3.1)	2.2 (1.5–3)	2.1 (1.5–2.9)	2.1 (1.5–2.8)	0.28

**Table 3:** Univariate and multivariate analysis of T cell subsets in association with recipients’ age, dialysis vintage (DV), and cold ischemia time (CIT).

	Univariate Regression					Multivariate Regression			
				95% Confidence Interval				95% Confidence Interval	
T cells	β	R^2^	*p*	lower	upper	β	*p*	lower	upper
Age	−0.41	0.17	<0.001	−29.8	−11.7	−0.32	0.001	−26.5	−6.6
DV	−0.29	0.08	0.002	−6.2	−1.4	−0.06	0.6	−3.9	2.3
CIT	−0.31	0.09	0.001	−38.2	−9.8	−0.14	0.23	−29.5	7.1
CD4+ T cells									
Age	−0.42	0.18	<0.001	−16.7	−6.8	−0.33	0.001	−14.8	−3.9
DV	−0.31	0.09	0.001	−3.5	−0.8	−0.08	0.5	−2.2	1.2
CIT	−0.32	0.1	0.001	−21.2	−5.6	−0.13	0.25	−15.8	4.2
CD8+ T cells									
Age	−0.21	0.04	0.03	−9.1	−0.5	−0.15	0.14	−8.09	1.1
DV	−0.16	0.03	0.08	−2	0.13				
CIT	−0.22	0.05	0.02	−14.01	−1.02	−0.16	0.12	−12.5	1.4

**Table 4:** Changes in the immune cell counts of patients with eGFR ≤ 50 and >50 mL/min/1.73 m^2^, from T0 to T12.

	eGFR ≤ 50	eGFR > 50
T cells T0	1200 (1000–1600)	1200 (1000–1600)
T cells T12	1600 (1200–1885)	1900 (1500–2400)
*p*	0.01	<0.001
CD4+ T cells T0	522 (364–827)	481 (386–704)
CD4+ T cells T12	628 (458–893)	857 (616–1122)
*p*	0.12	<0.001
CD8+ T cells T0	308 (182–387)	314 (227–421)
CD8+ T cells T12	467 (367–672)	542 (389–767)
*p*	<0.001	<0.001
Natural Killers T0	225 (152–307)	148 (103–332)
Natural Killers T12	195 (107–297)	198 (145–319)
*p*	0.14	0.04
Regulatory T cells T0	26 (14–38)	23 (15–32)
Regulatory T cells T12	26 (20–42)	34 (23–49)
*p*	0.44	<0.001

## Data Availability

Data will be made available by the authors upon request.

## Supplementary Materials

The following supporting information can be downloaded at: https://www.mdpi.com/article/10.3390/jcm13175093/s1, Table S1: Post hoc analysis of changes of T cell subset absolute count and proportions from T0 to T3, T6 and T12. Significance level is set to <0.008 after Bonferroni correction; Table S2: Post hoc analysis of changes of T cell subset absolute count and proportions from T3 to T6 and T12, as well as from T6 to T12. Significance level is set to <0.008 after Bonferroni correction; Table S3: Spearman correlation of T cell subset counts with recipients’ age, Dialysis vintage and cold ischemia time, at three (T3), six (T6) and twelve (T12) months post-transplantation.
