# Supplementary material for: Kidney Transplantation and Cellular Immunity Dynamics: Immune Cell Alterations and Association with Clinical and Laboratory Parameters
Source: J Clin Med. 2024 Aug 27;13(17):5093. doi: 10.3390/jcm13175093 (PMC11396483; doi:10.3390/jcm13175093)
Supplement: Supplementary file 1 [file jcm-13-05093-s001.zip › jcm-3135610-supplementary.pdf]

Supplemental Table S1. Post hoc analysis of changes of T cell subset absolute count and proportions from T0 to T3, T6 and T12. Significance level is set to <0.008 after Bonferroni correction.

T cell subsets	T0	T3	p
T cells (cells/μL)	1200 (1000 -1600)	1600 (1200-2300)	<0.001
T cells (%)	18.3 (14.8-24)	21.8 (16.8-31.5)	<0.001
CD4+ (cells/μL)	483 (384-707)	769 (526-1142)	<0.001
CD4+ (%)	41.4 (36.1-48.7)	47.4 (40.5-53.4)	<0.001
CD8+ (cells/μL)	310 (215-416)	437 (291-633)	<0.001
CD8+ (%)	24.6 (20.4-30.1)	26.9 (21.7-32)	0.011
NK cells (cells/μL)	210 (152-307)	151 (86-245)	<0.001
NK cells (%)	17.6 (11.8-25.2)	8.9 (5.4-14.9)	<0.001
Tregs (cells/μL)	24 (15-33)	29 (18-51)	<0.001
Tregs (%)	2.2 (1.4-3.1)	2.2 (1.5-3)	0.808
	T0	T6	p
T cells (cells/μL)	1200 (1000 -1600)	1700 (1300-2300)	<0.001
T cells (%)	18.3 (14.8-24)	24 (20.2-31.6)	<0.001
CD4+ (cells/μL)	483 (384-707)	807 (574-1116)	<0.001
CD4+ (%)	41.4 (36.1-48.7)	46.5 (40.3-53.4)	<0.001
CD8+ (cells/μL)	310 (215-416)	499 (341-648)	<0.001
CD8+ (%)	24.6 (20.4-30.1)	28.3 (24.1-34)	<0.001
NK cells (cells/μL)	210 (152-307)	150 (105-270)	0.026
NK cells (%)	17.6 (11.8-25.2)	9.2 (6.1-16.5)	<0.001
Tregs (cells/μL)	24 (15-33)	33 (24-48)	<0.001
Tregs (%)	2.2 (1.4-3.1)	2.1 (1.5-2.9)	0.461
	T0	T12	p
T cells (cells/μL)	1200 (1000 -1600)	1700 (1400-2300)	<0.001
T cells (%)	18.3 (14.8-24)	25.1 (20.3-31.6)	<0.001
CD4+ (cells/μL)	483 (384-707)	819 (600-1107)	<0.001
CD4+ (%)	41.4 (36.1-48.7)	46.8 (38.3-53.9)	0.004
CD8+ (cells/μL)	310 (215-416)	534 (387-743)	<0.001
CD8+ (%)	24.6 (20.4-30.1)	29.1 (24-38)	<0.001
NK cells (cells/μL)	210 (152-307)	150 (106-299)	0.015
NK cells (%)	17.6 (11.8-25.2)	8.7 (6-15.2)	<0.001

Tregs (cells/μL)	24 (15-33)	33 (21-48)	<0.001
Tregs (%)	2.2 (1.4-3.1)	2.1 (1.5-2.8)	0.490

Supplemental Table S2. Post hoc analysis of changes of T cell subset absolute count and proportions from T3 to T6 and T12, as well as from T6 to T12. Significance level is set to <0.008 after Bonferroni correction.

**T cell subsets**	**T3**	**T6**	**p**
T cells (cells/μL)	1600 (1200-2300)	1700 (1300-2300)	0.347
T cells (%)	21.8 (16.8-31.5)	24 (20.2-31.6)	0.007
$CD4^+$ (cells/μL)	769 (526-1142)	807 (574-1116)	0.68
$CD4^+$ (%)	47.4 (40.5-53.4)	46.5 (40.3-53.4)	0.201
$CD8^+$ (cells/μL)	437 (291-633)	499 (341-648)	0.082
$CD8^+$ (%)	26.9 (21.7-32)	28.3 (24.1-34)	0.018
NK cells (cells/μL)	151 (86-245)	150 (105-270)	0.022
NK cells (%)	8.9 (5.4-14.9)	9.2 (6.1-16.5)	0.028
Tregs (cells/μL)	29 (18-51)	33 (24-48)	0.156
Tregs (%)	2.2 (1.5-3)	2.1 (1.5-2.9)	1
	T3	T12	p
T cells (cells/μL)	1600 (1200-2300)	1700 (1400-2300)	0.261
T cells (%)	21.8 (16.8-31.5)	25.1 (20.3-31.6)	0.16
$CD4^+$ (cells/μL)	769 (526-1142)	819 (600-1107)	0.689
$CD4^+$ (%)	47.4 (40.5-53.4)	46.8 (38.3-53.9)	0.032
$CD8^+$ (cells/μL)	437 (291-633)	534 (387-743)	0.001
$CD8^+$ (%)	26.9 (21.7-32)	29.1 (24-38)	<0.001
NK cells (cells/μL)	151 (86-245)	150 (106-299)	0.072
NK cells (%)	8.9 (5.4-14.9)	8.7 (6-15.2)	0.084
Tregs (cells/μL)	29 (18-51)	33 (21-48)	0.439
Tregs (%)	2.2 (1.5-3)	2.1 (1.5-2.8)	0.845
	T6	T12	p
T cells (cells/μL)	1700 (1300-2300)	1700 (1400-2300)	0.225
T cells (%)	24 (20.2-31.6)	25.1 (20.3-31.6)	0.595
$CD4^+$ (cells/μL)	807 (574-1116)	819 (600-1107)	0.656
$CD4^+$ (%)	46.5 (40.3-53.4)	46.8 (38.3-53.9)	0.225

$CD8^+$ (cells/μL)	499 (341-648)	534 (387-743)	0.023
$CD8^+$ (%)	28.3 (24.1-34)	29.1 (24-38)	0.005
NK cells (cells/μL)	150 (105-270)	150 (106-299)	0.951
NK cells (%)	9.2 (6.1-16.5)	8.7 (6-15.2)	0.214
Tregs (cells/μL)	33 (24-48)	33 (21-48)	0.647
Tregs (%)	2.1 (1.5-2.9)	2.1 (1.5-2.8)	0.684

Supplemental Table S3. Spearman correlation of T cell subset counts with recipients' age, Dialysis vintage and cold ischemia time, at three (T3), six (T6) and twelve (T12) months post-transplantation.

	T cells (cells/μL)					
	T3		T6		T12	
	r	p	r	p	r	p
**Age at Tx**	-0.29	0.002	-0.23	0.02	-0.3	0.002
**Dialysis vintage**	-0.29	0.002	-0.2	0.04	-0.29	0.002
**Cold Ischemia Time**	-0.32	0.001	-0.15	0.13	-0.22	0.02
	**CD4 + T cells (cells/μL)**					
	**T3**		**T6**		**T12**	
	r	p	r	p	r	p
**Age at Tx**	-0.33	0.001	-0.29	0.002	-0.34	<0.001
**Dialysis vintage**	-0.36	<0.001	-0.27	0.005	-0.32	0.001
**Cold Ischemia Time**	-0.29	0.003	-0.17	0.07	-0.26	0.009
	**CD8+ T cells (cells/μL)**					
	**T3**		**T6**		**T12**	
	r	p	r	p	r	p
**Age at Tx**	-0.29	0.003	-0.1	0.31	-0.18	0.06
**Dialysis vintage**	-0.28	0.005	-0.1	0.26	0.17	0.08
**Cold Ischemia Time**	-0.36	<0.001	-0.16	0.09	-0.19	0.04
	**Tregs (cells/μL)**					
	**T3**		**T6**		**T12**	

	r	p	r	p	r	p
**Age at Tx**	-0.27	0.006	-0.286	0.004	-0.31	0.001
**Dialysis vintage**	-0.32	0.001	-0.24	0.01	-0.16	0.10
**Cold Ischemia Time**	-0.36	<0.001	-0.29	0.003	-0.1	0.29

	**NK cells (cells/μL)**					
	**T3**		**T6**		**T12**	
	r	p	r	p	r	p
**Age at Tx**	0.02	0.82	0.15	0.11	0.14	0.14
**Dialysis vintage**	-0.02	0.83	0.08	0.41	0.06	0.51
**Cold Ischemia Time**	-0.07	0.45	0.09	0.32	0.08	0.40
